# Myocardial skeletal muscle signal spoiling using a crusher coil: a human cardiac phosphorus (^31^P) MR spectroscopic imaging study at 7 Tesla

**DOI:** 10.1186/1532-429X-17-S1-P247

**Published:** 2015-02-03

**Authors:** Benoit Schaller, William T Clarke, Stefan Neubauer, Matthew D Robson, Chris Rodgers

**Affiliations:** Radcliffe Department of Medicine, Cardiovascular Medicine, Oxford Centre for Clinical Magnetic Resonance Research, Oxford, UK

## Background

^31^P-MRS provides direct insights into myocardial energy supply (ATP, ADP, phosphocreatine (PCr) and inorganic phosphate). An initial study demonstrated that 7T cardiac ^31^P-MRS has 2.8x greater SNR than at 3T. However, the translation of more sophisticated ^31^P-MRS protocols to 7T is particularly challenged by increased RF heating of tissue at 7T. Chen and Ackerman introduced the surface spoiling coil in 1990: a concept that was recently further developed (Boer MRM 2014) for lipid suppression in human brain ^1^H-CSI. In this work, we introduce the first crusher coil for cardiac ^31^P-MRS at 7T. This allows us to saturate more efficiently skeletal muscle signal removing the RF heating associated with RF saturation bands.

## Methods

Data were acquired with a Siemens 7T scanner. Localization used a 10cm ^1^H Tx/Rx RF coil (Rapid Biomedical) to acquire CINE FLASH images. ^31^P-MR spectra were acquired with a custom 10cm ^31^P Tx/Rx loop. The magnetic field generated by the crusher coil was simulated and optimized using Matlab (Mathworks). A capacitor initially charged by a power supply unit (PSU) was used to drive the current pulse in the crusher coil during a short spoiling duration (100μs). Spoiling was timed to coincide with the existing phase encoding gradients. A 2D-CSI experiment was performed on a two-compartment phantom with the ^31^P RF coil and the crusher coil placed above it. The BISTRO saturation band (Luo, MRM 2001) covered the entire top slice. The spoiling efficiency was then confirmed in vivo using 3D-CSI.

## Results

**Figure 1:**
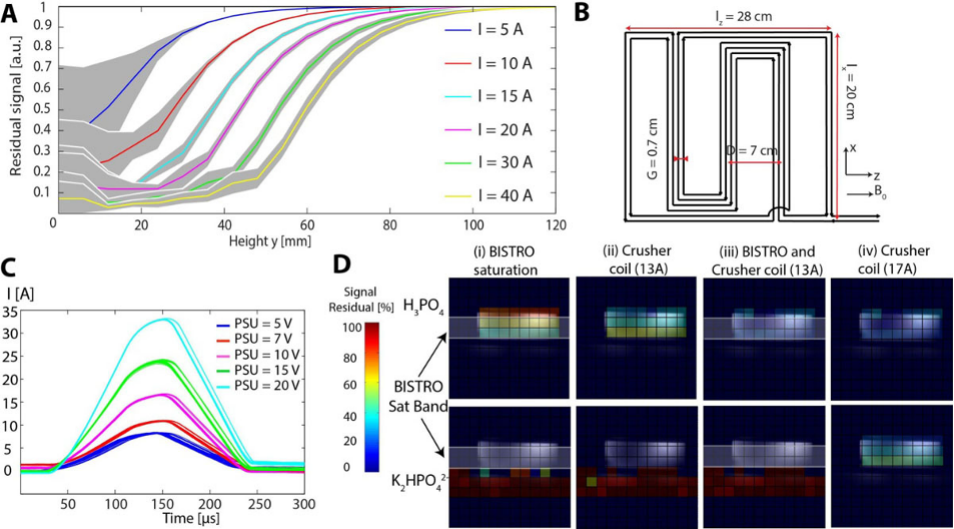
(A) Simulation of the residual signal for different current (T_spoil_=100 μs). (B) Dimensions of the crusher coil. (C) Measured current flowing through the crusher coil for different drive voltages. (D) 2D-CSI in vitro experiment (TR = 1 s, TE = 2.3 ms, slice thickness=20 mm, matrix size = 180×180 mm^2^, res = 12×12, over a transversal slice) with a 2 slice phantom (top: H_3_PO_4_ and bottom: K_2_HPO_4_^2-^) with 5 saturation protocols: (i) BISTRO saturation bands (thickness = 30mm), (ii) crusher spoiling (equivalent area to I_spoil_×T_spoil_= 13×100 A.μs square pulse), (iii) BISTRO and crusher spoiling (I_spoil_×T_spoil_= 13×100 A.μs) and (iv) crusher spoiling (I_spoil_×T_spoil_= 17×100 A.μs). The residual signal was obtained by normalizing the signal in each case by the unsuppressed signal, for each voxel in turn.

**Figure 2:**
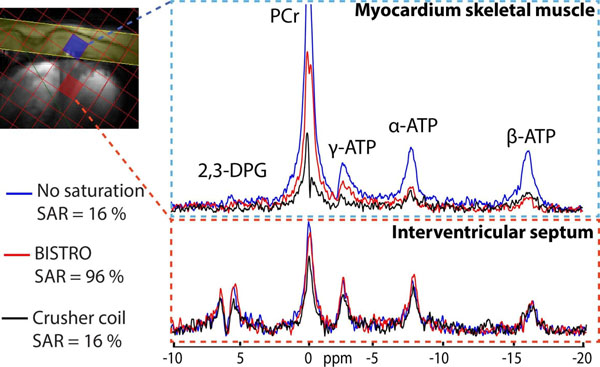
^31^P-MR: spectra acquired in the skeletal muscle and in the interventricular septum for different saturation protocols: no saturation (blue spectra), BISTRO saturation band (red spectra) and crusher coil (black spectra). Parameters of 3D CSI: TR = 1 s, TE = 2.3 ms, matrix size = 240×240×200 mm^3^, resolution = 16×16×8, 10 averages, TA = 28 min. Parameters of the crusher coil: I_spoil_ = 5 A and T_spoil_ = 100 μs. Inset: CSI grid overlaid on anatomical image with voxel positions in skeletal muscle (blue voxel) and in the interventricular septum (red voxel). BISTRO saturation band is illustrated as a yellow band in the inset.

The coil geometry was optimized to saturate skeletal muscle (<40mm) with minimal disruption of cardiac signals (>70mm) (Fig. 1A-B). Currents up to 35A were produced through the crusher coil (Fig. 1C). In the 2D CSI *in vitro* experiment (Fig. 1D), the bottom slice signal remained stable, while the top slice signal was spoiled differently depending on the acquisition protocol. The SAR-limited BISTRO saturation bands reduced the mean signal in the entire top slice to 52% of the original signal. A similar signal reduction (45%) occurred when using the crusher coil at 13A. When combining the BISTRO saturation bands and the crusher coil (13A), the mean signal was reduced to 15%. The current in the crusher coil was increased up to 17A, yielding to a 35% signal reduction. In 3D-CSI *in vivo* study, mean skeletal muscle signal was reduced to 52% with BISTRO and to 40% with the crusher coil (Fig. 2). The PCr/ATP ratio in the septum was 2.3 (BISTRO) and 2.1 (crusher). SAR was 97% (BISTRO) and 16% (crusher).

## Conclusions

A crusher coil is an efficient alternative to BISTRO saturation bands for suppressing skeletal muscle during cardiac ^31^P-MRS at 7T. The flexibility offered by using the crusher coil will allow us to employ sequence modules that would otherwise be SAR-prohibitive e.g. adiabatic excitation for absolute quantitation, ^1^H-^31^P NOE enhancement or saturation-transfer pulses for future clinical studies at 7T, without having to compromise the skeletal muscle suppression.

## Funding

This study was supported by a Sir Henry Dale Fellowship from the Wellcome trust and the Royal Society [098436/Z/12/Z].

